# Fermentation Characteristics of *Lactococcus lactis* subsp. *lactis* Isolated From Naturally Fermented Dairy Products and Screening of Potential Starter Isolates

**DOI:** 10.3389/fmicb.2020.01794

**Published:** 2020-08-04

**Authors:** Weicheng Li, Min Ren, Lana Duo, Jing Li, Shuai Wang, Yaru Sun, Min Li, Weiyi Ren, Qiangchuan Hou, Jie Yu, Zhihong Sun, Tiansong Sun

**Affiliations:** ^1^Key Laboratory of Dairy Biotechnology and Engineering, Ministry of Education, Inner Mongolia Agricultural University, Hohhot, China; ^2^Key Laboratory of Dairy Products Processing, Ministry of Agriculture and Rural Affairs, Inner Mongolia Agricultural University, Hohhot, China; ^3^Key Laboratory of Dairy Biotechnology and Engineering, Inner Mongolia Agricultural University, Hohhot, China

**Keywords:** *Lactococcus lactis* subsp. *lactis*, fermentation characteristics, potential starter culture, malt aroma, large-scale phenotyping

## Abstract

It is well known that consumers are keen to try fermented milk products with different flavors and starter cultures are important in determining the resulting fermented dairy products. Here, we present the phenome of 227 *Lactococcus lactis* subsp. *lactis* isolates from traditionally fermented dairy products and the selection of potential starter strains. Large-scale phenotyping revealed significant technological diversity in fermentation characteristics amongst the isolates including variation in fermentation time, viscosity, water holding capacity (WHC) and free amino nitrogen (FAN) production. The 16 isolates with the best fermentation characteristics were compared, in a sensory evaluation, with the commercial starter Chr. Hansen R-704 as excellent fermentation characteristics to identify potential starter isolates and find the isolate which can product good flavors. From these, and from solid phase micro extraction (SPME) – gas chromatography (GC)-mass spectrometry (MS) analysis, we identified IMAU11823 and IMAU11919 as producing 3-methyl butanal and 3-methyl-2-butanone which contribute to the malt aroma. This study expands the characterization of *L. lactis* subsp. *lactis* phenotypic dataset and technological diversity and identified isolates with potential culture starter in the fermentation industry.

## Introduction

People from different cultural backgrounds, and from across the globe, have been consuming fermented dairy products for millennia. Traditionally fermented dairy products are considered, not just as food, but also as a nutritionally complete food with a number of health benefits (Dehghan et al., 2018). These and other health-promoting features, combined with the view that fermented foods are “natural,” has further increased the popularity of fermented dairy foods (Kumar et al., 2015) including yogurt, kefir, dairy fan (resembling cheese), dairy cake, koumiss, sour cream, butter, and cheese. Natural fermented dairy products are rich in a diversity of lactic acid bacteria (LAB). LAB with different functional attributes are excellent microbial resources for industrial production, especially in the dairy industry (Cretenet et al., 2011).

As primary components of the starter cultures used in fermented food production, members of the LAB group are of key industrial importance (McAuliffe, 2018). As a typical species of *Lactococcus* (*L.*) which is famous genera in LAB, *Lactococcus lactis* is widely used in industry. Isolates of *L. lactis* subsp. *lactis* are generally fast acidifiers, while isolates of *L. lactis* subsp. *cremoris* are often favored as defined starters because they tend to cause less bitterness. In addition to the widely recognized applications of *L. lactis* subsp. *lactis* in cheese and butter production (Madera et al., 2003; Özkalp et al., 2007), its special odor-providing attributes are also important to improve the quality of fermented milk products (especially low-temperature fermented milk) (Omaea et al., 2008; Cavanagh et al., 2015). The *L. lactis* subsp. *lactis* isolates used as starter cultures for commercial production have numerous isolate-dependent characteristics such as lactose fermentation capabilities, proteolytic activity, exopolysaccharide (EPS) production, and flavor production; they also play an essential role in the formation of aroma, texture and acidity in the final products (McAuliffe, 2018). Chr. Hansen R-704 was a commonly used starter cultures in China. Although it cannot produce special fermentation aroma, it has useful and excellent fermentation characteristics. Therefore, Chr. Hansen R-704 was used in this study as a reference strain for screening potential starter strains.

Flavor characteristics of the milk to be used for fermentation are also of primary importance for the quality of the final fermented product (Gallardo-Escamilla et al., 2005). The dairy products market is continually expanding, and consumers are increasingly experimenting with different flavors and ingredients. Thus, dairy companies are always striving to ensure their products have unique characteristics that set them apart from others. To achieve this, many companies have investigated culture manipulation as a tool for flavor diversification (McAuliffe, 2018). When screening starter cultures, it is important to consider the aroma-producing capacity of isolates alongside the usual fermentation characteristics.

Recently, there has been increased interest in exploring the production of flavor-producing compounds by isolates from different natural ecosystems. Formation of volatile compounds is key to flavor enhancement; production of these compounds is as a result of glycolysis, lipolysis, citrate metabolism and particularly proteolysis of amino acids (Rvan et al., 2002; Gerrit et al., 2010). Isolates of *L. lactis* subsp. *lactis* biovar *diacetylactis* ferment citrate, which contributes to flavor and aroma via the production of diacetyl. The decarboxylation activity of non-dairy isolates, in monoculture and in co-culture with industrial dairy isolates, was evaluated by Ayad et al. (2010). They found that some non-dairy isolates in monoculture had strong decarboxylation activity against α-ketoisocaproic acid as indicated by high levels of the malt odor compound 3-methyl butanal in cell-free extracts. The compound 3-methyl butanal, and also 3-methyl-2-butanone, can endow fermented dairy products with different flavors including: fresh malt, nutty, floral, and fruity flavors (Jensen et al., 1994; Adhikari et al., 2010; Galvão et al., 2011). Seventy-eight percent of Chinese consumers prefer “nutty malt flavors,” such as those that are associated with mature cheese (Zhang et al., 2011). Therefore, the development of nutty and malt flavors by starter cultures is particularly suited to Chinese consumers.

Looking for strains that have good fermentation properties and produce specific aromas can be of great help not only in industrial production, but also in understanding the main components of these aromas. The present study aimed to identify the fermentation characteristics of wild-type *L. lactis* subsp. *lactis* isolates from naturally fermented dairy products and screen for those with greatest potential as starter cultures because they good fermentation characteristics. We recorded fermentation characteristics of isolates during both the fermentation stage and the storage stage at 4°C and used the results to select isolates with favorable attributes for comparison with a commercial starter Chr. Hansen R-704. And we used sensory evaluation to identify those with the ability to produce a malt odor. Solid phase micro extraction (SPME)-gas chromatography (GC)-mass spectrometry (MS) was used to compare the volatile flavor profiles of four isolates that either produced fermented milk with good flavor, or without good flavor. Our work aimed to provide a large-scale phenomics data for isolates with different metabolic activities, as demanded by the dairy industry in order to expand their product portfolio. In addition, we also identified two isolates that produced the malt odor and had the greatest potential as starter cultures.

## Materials and Methods

### Experimental Design

In the first fermentation run we made an initial characterization of the fermentation attributes of 227 *L. lactis* subsp. *lactis* isolates from naturally fermented dairy products, in whole milk at 30°C. The fermented milk from those isolates that had achieved fermentation of whole milk in less than 32 h (*n* = 190) were stored at 4°C and their acid production properties assessed. From these isolates the ones that fermented milk within 12 h and had good acidification profiles during storage (*n* = 40) were identified and their combined fermentation and storage properties in the first fermentation run reconsidered. In this way isolates with suboptimal attributes were excluded leaving a subset (*n* = 24) to be taken forward for more detailed comparison with the commercial starter, Chr. Hansen R-704, in a second fermentation run. Chr. Hansen R-704 was used as a benchmark to compare the technological characteristics (e.g., TA, pH, water holding capacity). Results from the second fermentation run allowed selection of isolates with the greatest potential (*n* = 16). These isolates were then used to produce fermented milk for sensory evaluation, in comparison with the commercial starter, to identify those that also had special flavors. Finally, we used SPME-GC-MS to compare differences in volatile flavor profiles amongst two isolates with the best sensory profiles (which produced the special flavors) and two other isolates that did not produce the special flavors.

### *Lactococcus lactis* subsp. *lactis* Isolates and Reagents

The 227 isolates of *L. lactis* subsp. *lactis* used in this study were from the Lactic Acid Bacteria Collection Center (LABCC) of the Inner Mongolia Agricultural University, Hohhot, China (Supplementary Table S1). The isolates had been isolated from a variety of traditionally fermented foods, specifically from: shubat (ten isolates), yogurts (111 isolates), dairy cake (26 isolates), acid porridge (one isolate), sour cream (ten isolates), koumiss (seven isolates), dried milk cake (one isolate), milk (nine isolates), cheese (four isolates), goats’ yogurt (four isolates), goats’ milk (two isolates), fan acid whey (one isolate), and jiaoke (41 isolates); a map showing the geographical locations from which isolates came is shown in Supplementary Figure S1. They were identified as *L. lactis* using a combination of traditional microbial identification methods in combination with 16S ribosomal RNA (rRNA) gene sequence analysis (Bao et al., 2010). 16S rRNA gene sequences were submitted to NCBI GenBank and isolate-specific information is shown in Supplementary Table S1. All isolates were stored long-term in a skimmed milk medium (SMM, NZMP LTD., New Zealand) at −80°C.

### Activation, Multiplication, and Viable Counts of Bacterial Isolates

To activate the 227 *L. lactis* subsp. *lactis* isolates from the culture collection, they were each cultured in skimmed milk medium at 37°C for 24 h. After this, the culture was multiplied over two generations in M17 (OXOID, London, United Kingdom) liquid medium with 2% inoculation volume (Terzaghi and Sandine, 1975). The bacteria were collected by centrifugation at 3500 r/min for 15 min, washed twice in phosphate-buffered saline (PBS), and then diluted to concentrations of 10^7^ and 10^8^ colony forming units (CFUs) per mL by PBS. After a further 48 h at 37°C the cells were counted using the pouring method and suspensions placed at 4°C prior to use in fermentation runs (Li et al., 2018).

### Preparation of Fermented Milks

The fermented milk was prepared based on the National Standard of the People’s Republic of China (2010; standard GB 19302–2010) and a previously published work (Wang et al., 2010). The reconstituted milk was prepared from 11.5% (wt/wt) whole milk powder (NZMP Co. Ltd., Wellington, New Zealand) (containing, per 100 g, 39.1 g of lactose, 26.8 g of fat, and 25.0 g of protein) with the addition of 6.5% sucrose. The whole milk powder and sucrose were blended with sterilized distilled water at 65°C and homogenized at 20 MPa by using a high-pressure homogenizer (SRH, Shenlu, Shanghai) before pasteurization (95°C for 5 min). The whole milk was then heated at 95°C for 5 min to pasteurize it and then cooled to 30°C. In all fermentation runs the number of viable bacterial cells were enumerated for each isolate and inoculated into whole milk to achieve a dose of 1 × 10^6^ CFU/mL. The fermentation experiment was performed in triplicate. Fermentation was done at 30°C until each culture suspension achieved a pH of 4.6. The time taken to achieve this was recorded. In the first fermentation run the fermented milk produced by isolates that had achieved a pH of 4.6 within 32 h (*n* = 190) was then stored at 4°C for 24 h, the same amount of time as commercially produced fermented milk is stored in China.

### Determination of Fermentation Characteristics of Isolates

In the first fermentation run samples of fermented milk were taken 0, 12, 16, 20, 24, 28, and 32 h following the inoculation of the starter cultures. The pH, titratable acidity (TA, °T) and free amino nitrogen (FAN) were determined in triplicate for each sample. The TA and pH of the fermented milk were determined based on standard methods described by the Association of Official Analytical Chemists (AOAC, 2000). The FAN content was determined based on methods described by Church et al. (1983).

### Determination of Acidification Characteristics of Isolates During Storage

During the 24 h storage period of fermented milks from the first fermentation run, additional samples were taken at 0 (i.e., the time that each isolate achieved pH 4.6), 12 and 24 h. The pH, TA, viscosity, water holding capacity (WHC), and FAN content were determined. Viscosity was determined using a viscometer (BROOKFIELD, Middleboro, MA, United States). WHC was determined using previously described methods (Farooq and Haque, 1992).

### Sensory Evaluation

Isolates selected (*n* = 16) for sensory profiling were compared with the commercial starter, Chr. Hansen R-704 (Hansen Co., LTD., Hoersholm, Denmark) which compounded by several *L. lactis* strains. Each isolate was used as a starter culture to produce fermented milk as described previously and then submitted to sensory analysis using the methods of Bayarri et al. (2011). The scoring standards are shown in Supplementary Table S2. Participants were asked to taste all the fermented milks and drink water between each sample to cleanse the palate. Scores are presented as mean ± standard deviations (SD). The review team was graded according to the scoring criteria and identified the number of strains with different flavors from commercial starter products.

### Volatile Flavor Profiles

We used SPME-GC-MS (Agilent 7890B gas chromatograph; Agilent Technologies Inc., Palo Alto, CA, United States) to detect differences in the volatile flavor profiles of the two isolates with the best sensory evaluation scores and the two isolates with the worst sensory evaluation scores. Volatile compounds from the fermented milk were identified using a 7890B gas chromatograph equipped with a 5977A mass selective detector (Agilent Technologies Inc., Palo Alto, CA, United States). Volatile compounds adsorbed onto the SPME fibers (Supelco, Bellefonte, PA, United States), were passed through an HP-5MS column (30 m length, 0.25 mm i.d., 0.25 μm film thickness, Agilent Technologies Inc.).

Twenty milliliters fermented milk were placed in 100 mL gas washing flask with a purge head, and 1 μL 1,2-Dichlorobenzene solution (Sigma-Aldrich, St. Louis, MO, United States) was added as internal standard solution. The final concentration of internal standard solution in each sample was 10 μg/L. All samples were analyzed in triplicate by SPME-GC-MS. The following methods were used at each step of detection.

*SPME conditions:* The products were stirred with micro-stir bars for 60 min at 50°C to allow the samples to reach equilibrium with magnetic stirring (400 rpm). Fibers were then inserted into the GC-MS injector port for desorption (3 min) at 270°C to desorb volatile compounds into the gas chromatograph.

*GC conditions:* Helium was used as the carrier gas at 1 mL/min. The gas chromatograph temperature was maintained at 35°C for the first 5 min, then increased to 140°C at a rate of 5°C/min for 2 min, and gradually increased to 250°C at a rate of 10°C/min for the final 3 min (Dan et al., 2017).

*MS conditions:* The MS detector was operated at 150°C in electron impact mode at a voltage of 70 eV and had an ion source temperature of 230°C. The mass spectra of the treated samples were recorded within a mass range of 35–500 m/z with five scans and no solvent delay.

Volatile compounds were identified by matching of their mass spectra with those of commercial database (National Institute of Standards Technology Mass Spectral Database) and confirmed by comparison with retention indices (RI) of authentic reference compounds or RI recorded in the literature. The relative abundances of all identified volatile compounds were semi-quantified as peak areas in total ion chromatography (TIC).

### Statistical Analysis

All experimental data were analyzed using R software (ver. 3.5.0). Bar plots were constructed using the “ggplot2” package. Correlations were analyzed and plotted using the “ggcorrplot” package. The heatmap was plotted using the “pheatmap” package. The sensory evaluation scores were presented using the Microsoft Excel 2016.

## Results

### Fermentation Characteristics of *L. lactis* subsp. *lactis* Isolates During Fermentation and Acid Production Properties During Storage

**FIGURE 1:**
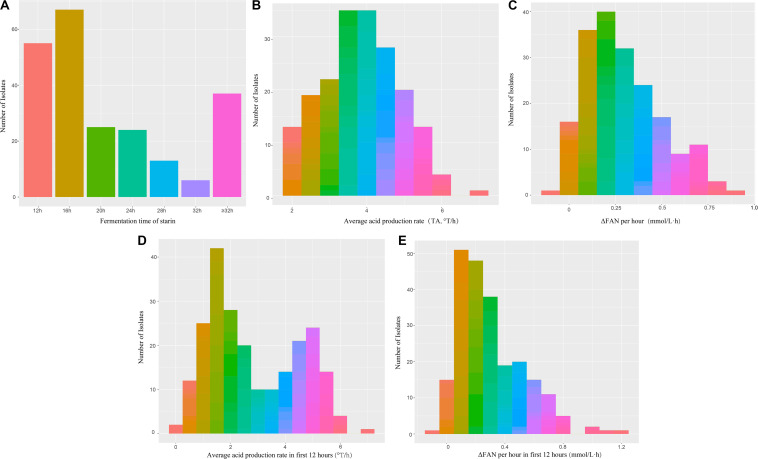
Frequency histogram of fermentation capacity of 227 isolates of *L. lactis* subsp. *lactis* during fermentation **(A)** Fermentation time (hours); **(B)** Average acid production rate (°T/h); **(C)** Average protein production rate (mmol/L⋅h); **(D)** Average acid production rate in first 12 h (°T/h); **(E)** Average protein production rate per hour in first 12 h (mmol/L⋅h).

The fermentation characteristics of the 227 *L. lactis* subsp. *lactis* isolates from traditionally fermented dairy products were determined. The fermentation times (i.e., time to achieve pH 4.5) were 12, 16, 20, 24, and 28–32 h for 55, 67, 25, 24, and 19 isolates (190 in total), respectively (Figure 1). There were 37 isolates that did not finish fermentation within 32 h and these isolates were not evaluated during storage.

**FIGURE 2:**
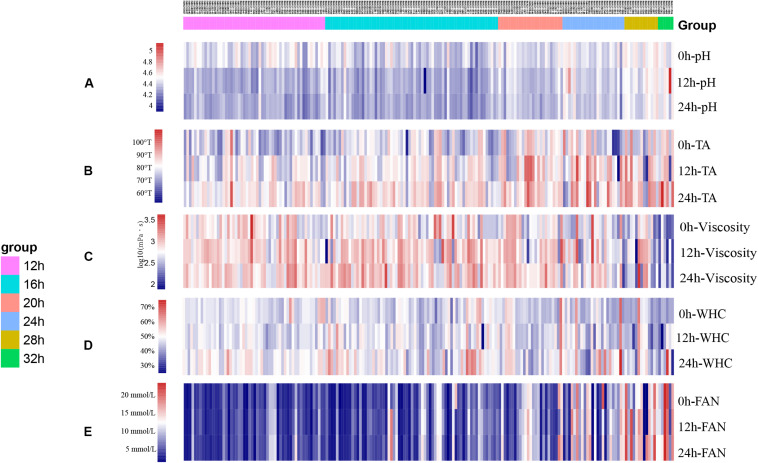
The heatmap of fermentation capacity of 227 isolates of *L. lactis* subsp. *lactis* during storage at 4°C **(A)** pH; **(B)** TA (°T); **(C)** Viscosity (mPa⋅s); **(D)** WHC; **(E)** FAN (mmol/L).

Variation in acidification capacity and proteolytic capacity, during storage of isolates that completed fermentation within 32 h (*n* = 190) can be seen in Figures 2B,C. The acidification capacity of half of the isolates was lower than 4.0°T/h; isolate IMAU11060 had the best (greatest) acidification capacity at 6.88°T/h. IMAU20795 and IMAU11468 had the best ΔFAN per hour at 0.682 and 0.639 mmol/L⋅h, respectively.

Acidification capacities of the 190 isolates between 0 and 12 h was bimodal with the first peak having a value of 1.2°T/h and the second peak a value of 5°T/h (Figure 1D). The values of ΔFAN per hour for most isolates were between 0 and 0.4 mmol/L⋅h (Figure 1E).

The pH, TA, viscosity, WHC, and FAN of the 190 isolates after 0, 12, and 24 h of storage can be seen in Figures 2A–E, respectively. Isolates that had the same fermentation times showed similar trends in variability (Figure 2). Overall, the pH of most isolates decreased while the TA increased during storage. Isolates with weak post-fermentation acidification characteristics were selected for further evaluation.

Of the 190 isolates tested, 44 had good viscosity-producing properties with viscosities exceeding 900 mPa⋅s after 24 h storage. Thirty-four isolates had viscosities below 300 mPa⋅s. We found that 26 of the 190 *L. lactis* subsp. *lactis* isolates had WHCs greater than 50% (considered as high quality) while most (155 of 190) had WHC values between 40 and 50%. Most isolates had low FAN values during storage.

### Correlation Analysis

**FIGURE 3:**
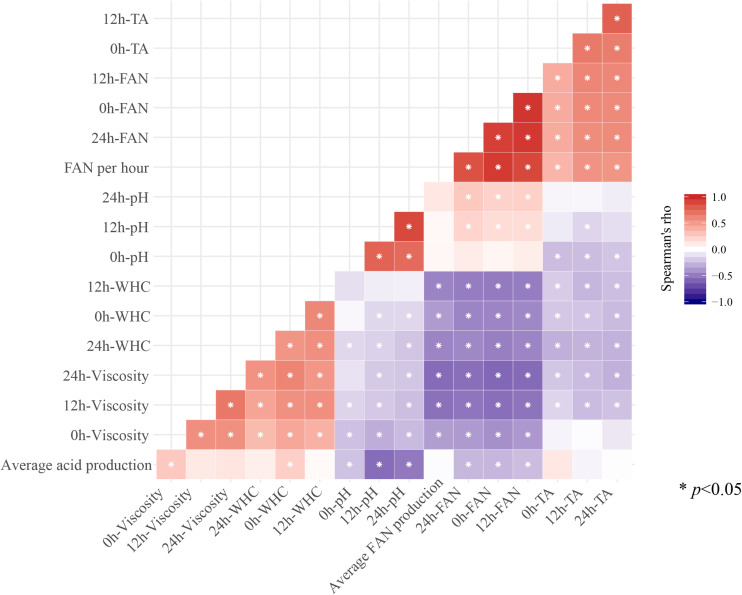
The correlation between fermentation characteristic index and fermentation characteristic index during storage (^∗^ means *p* < 0.05).

We further analyzed correlations amongst the mean values for all the indicators measured during fermentation and storage. Most indicators were significantly correlated with each other (*p* < 0.05; Figure 3). For instance, ΔFAN per hour during fermentation was positively correlated with FAN and TA during storage (*r* > 0; *p* < 0.05). However, ΔFAN per hour during fermentation was negatively correlated with WHC and viscosity during storage (*r* < 0; *p* < 0.05). Additionally, there were positive correlations between FAN and TA during storage, as well as between WHC and viscosity (*r* > 0; *p* < 0.05). Conversely, FAN, TA, WHC, and viscosity were negatively correlations with each other (*r* < 0; *p* < 0.05). The TA per hour during fermentation was negatively correlated with pH and FAN during storage (*r* < 0; *p* < 0.05). In addition, the TA per hour during fermentation was positively correlated with pH and FAN during storage (*r* < 0; *p* < 0.05).

### Selecting Isolates With Greatest Potential as Starter Cultures

Of the 40 isolates that completed milk fermentation within 12 h and had a strong acid production capacity (Table 1) acid production of isolates IMAU40066, IMAU90233, IMAU96006, IMAU11161, IMAU10854, and IMAU10941 increased to more than 20°T during storage which is not optimal and so they were excluded from further evaluation. During storage the viscosity, WHC and FAN of fermented milk made by IMAU32258, IMAU10940, IMAU96004, IMAU32070, IMAU11885, IMAU10422, IMAU10850, IMAU10937, IMAU11049, IMAU11882, and IMAU90221 (total 11 isolates) increased and then decreased; this instability meant they too were excluded. This left 24 isolates for comparison with the commercial isolate, Chr. Hansen R-704, in the second fermentation run.

**TABLE 1:** The acid production rate of 40 isolates with good fermentation rate at first screening.

Isolate number	Acid production rate	Acid production quantity	Isolate number	Acid production rate	Acid production quantity
	(Δ°T/h)	(°T)		(Δ°T/h)	(°T)
IMAU10066	4.60	55.08	IMAU11863	5.14	63.25
IMAU10407	5.17	55.88	IMAU11879	5.09	58.19
IMAU10422	5.78	52.90	IMAU11882	5.00	63.10
IMAU10850	5.03	60.36	IMAU11885	5.26	70.11
IMAU10854	5.27	66.86	IMAU11886	5.03	61.15
IMAU10937	5.18	67.61	IMAU11905	5.43	62.09
IMAU10940	4.99	53.72	IMAU11906	5.57	65.49
IMAU10941	5.22	62.19	IMAU11919	4.34	63.32
IMAU10982	5.11	54.34	IMAU11951	5.28	49.82
IMAU10987	5.63	51.05	IMAU32070	5.63	59.64
IMAU11049	4.64	61.73	IMAU32258	4.99	65.55
IMAU11161	4.48	61.30	IMAU40066	4.66	59.85
IMAU11411	5.46	60.00	IMAU50105	4.15	50.01
IMAU11497	5.98	55.66	IMAU50117	4.17	67.54
IMAU11546	4.41	65.20	IMAU90221	4.78	60.50
IMAU11547	5.04	55.15	IMAU90233	4.97	71.78
IMAU11820	4.59	57.11	IMAU92143	5.26	57.39
IMAU11822	4.53	63.18	IMAU94238	4.76	62.60
IMAU11823	4.85	52.06	IMAU96004	5.46	59.85
IMAU11838	5.10	61.08	IMAU96006	4.25	69.38

In the second fermentation run we compared the fermentation capacity of the 24 isolates with the commercial starter to identify those with greatest potential. The fermentation time of the experimental isolates was between 7.59 and 20.76 h, while the fermentation time of the commercial isolate, R-704, was 7.66 h (Table 2). The fermentation time of IMAU92143 and IMAU90233 was 7.59 h and 7.65 h, respectively, and they also had a strong acid production capacity. There were 18 isolates with longer fermentation times than Chr. Hansen R-704, although they were still less than 12 h, which was consistent with the results of the first fermentation run. However, isolates IMAU11863, IMAU11905, IMAU11497, IMAU11951, IMAU11820, IMAU10066, IMAU11547, and IMAU11886 took longer than 16 h, and the texture of the fermented milk was poor; this indicated that these eight isolates had poor fermentation stability and would not be good starter cultures.

**TABLE 2:** The fermentation time of 24 isolates with good fermentation capacity and commercial starter at second screening.

Isolate number	Fermentation time (h)	Isolate number	Fermentation time (h)
R-704	7.66 ± 0.01	IMAU11863	18.76 ± 0.03
IMAU10066	19.54 ± 0.00	IMAU11879	7.85 ± 0.00
IMAU10407	9.75 ± 0.00	IMAU11885	8.13 ± 0.01
IMAU10982	11.17 ± 0.00	IMAU11886	20.76 ± 0.05
IMAU10987	11.25 ± 0.02	IMAU11905	20.34 ± 0.05
IMAU11411	7.74 ± 0.00	IMAU11906	8.09 ± 0.01
IMAU11497	16.39 ± 0.00	IMAU11919	7.78 ± 0.00
IMAU11546	12.11 ± 0.00	IMAU11951	20.59 ± 0.00
IMAU11547	19.51 ± 0.05	IMAU50105	11.11 ± 0.00
IMAU11820	19.51 ± 0.05	IMAU50117	13.09 ± 0.01
IMAU11822	7.88 ± 0.01	IMAU92143	7.59 ± 0.02
IMAU11823	7.89 ± 0.00	IMAU94238	8.91 ± 0.00
IMAU11838	8.17 ± 0.01		

### Screening Potential Starter Culture Isolates for Good Sensory and Odor Properties

From the comprehensive evaluation described above, 16 isolates were selected for sensory evaluation against the commercial isolate Chr. Hansen R-704, and they had the following attributes: fast acid production, weak post-fermentation acidification, good viscosity, good water-holding capacity and good proteolytic capacity. These isolates were IMAU92143, IMAU11411, IMAU11919, IMAU11879, IMAU11822, IMAU11823, IMAU11906, IMAU11885, IMAU11838, IMAU94238, IMAU10407, IMAU50105, IMAU10982, IMAU10987, IMAU11546, and IMAU50117.

The highest sensory evaluation score was 83 and achieved by Chr. Hansen R-704-fermented milk (Table 3), and the lowest was 58.71, achieved by IMAU10987-fermented milk. The overall sensory evaluation score of fermented milk produced by isolates IMAU11823, IMAU11906, and IMAU11919 was more than 80 points, and there was no significant difference between them and isolate Chr. Hansen R-704 (*p* > 0.05), showing they had good sensory qualities. The sensory evaluation scores of fermented milks from the other isolates were all higher than 60. Fermented milk made by isolates IMAU11919 and IMAU11823 had the best odor, which was identified as a malt odor. According to the sensory evaluation results, fermented milk made by isolates IMAU11823 and IMAU11919 not only has good fermentation characteristics and sensory characteristics, but also has a special flavor. These two isolates are used in SPME-GC-MS evaluation.

**TABLE 3:** The sensory evaluation of 16 isolates with good fermentation capacity and commercial starter at second screening.

Isolate number	Odor	Flavor	Appearance	Taste	Total score
R-704	22.86 ± 2.97	24.71 ± 2.63	18.57 ± 0.98	16.86 ± 1.95	83.00 ± 4.16^*A*^
IMAU10407	20.43 ± 5.29	21.14 ± 1.46	14.00 ± 3.65	12.43 ± 1.62	68.00 ± 8.60^*C*^
IMAU10982	23.71 ± 3.86	23.29 ± 3.25	17.29 ± 2.06	15.29 ± 1.25	79.57 ± 8.34^*AB*^
IMAU10987	17.43 ± 3.82	18.29 ± 4.50	9.86 ± 2.54	13.14 ± 2.61	58.71 ± 10.83^*D*^
IMAU11411	18.14 ± 3.80	17.43 ± 4.39	18.29 ± 1.25	14.29 ± 4.57	68.14 ± 11.63^*C*^
IMAU11546	21.14 ± 3.58	22.29 ± 3.99	9.86 ± 3.48	15.29 ± 1.89	68.57 ± 8.66^*C*^
IMAU11822	19.71 ± 3.35	21.71 ± 4.64	18.29 ± 1.25	15.43 ± 2.76	75.14 ± 8.84^*ABC*^
IMAU11823	22.71 ± 3.30	23.29 ± 4.07	18.43 ± 1.13	17.14 ± 1.68	81.57 ± 7.55^*AB*^
IMAU11838	19.29 ± 2.06	20.57 ± 3.91	18.14 ± 1.07	15.86 ± 1.95	73.86 ± 4.85^*ABC*^
IMAU11879	21.86 ± 1.46	24.00 ± 3.56	16.86 ± 2.97	15.00 ± 2.58	77.71 ± 8.06^*ABC*^
IMAU11885	22.71 ± 1.60	23.00 ± 2.38	16.50 ± 2.06	16.71 ± 2.81	78.93 ± 3.66^*AB*^
IMAU11906	23.57 ± 2.37	24.86 ± 3.58	15.71 ± 2.14	16.29 ± 2.29	80.43 ± 7.91^*AB*^
IMAU11919	25.14 ± 1.86	23.71 ± 2.50	17.07 ± 1.10	16.43 ± 3.26	82.36 ± 4.59^*A*^
IMAU50105	21.86 ± 3.93	20.71 ± 2.56	16.14 ± 1.86	14.71 ± 1.89	73.43 ± 7.98^*ABC*^
IMAU50117	20.86 ± 2.85	21.57 ± 2.44	15.43 ± 2.30	14.14 ± 1.46	72.00 ± 3.87^*C*^
IMAU92143	22.00 ± 3.11	21.43 ± 2.15	15.71 ± 3.45	14.29 ± 1.98	73.43 ± 8.58^*ABC*^
IMAU94238	20.86 ± 3.93	23.29 ± 2.81	15.00 ± 2.65	15.00 ± 2.38	74.14 ± 8.41^*ABC*^

*Different letters ^*A*−*D*^indicated significant differences in sensory scores of different strains (*P* < 0.05).*

### Identification of Volatile Flavors in Isolates That Produced a Malt Odor

Using SPME-GC-MS, a total of 54 volatile flavor compounds were detected in the fermented milk produced by the four selected isolates (IMAU11823, IMAU11919, IMAU10987, and IMAU10407), which included 11 acids, five aldehydes, seven ketones, ten alcohols, two esters, eight hydrocarbons, and seven other compounds (Table 4). The flavor substances of the fermented milk from the two groups (with or without malt odor) were similar, but some attributes were different. It is worth noting that 3-methyl-butanol and 3-methyl-2-butanone were present in larger quantities in fermented milk from isolates IMAU11823 and IMAU11919; 123.94 μg/L and 76.21 μg/L, respectively, for isolate IMAU11823, and 101.91 μg/L and 80.76 μg/L, respectively, for isolate IMAU11919.

**TABLE 4:** Volatile flavors in fermented milk produced by pure cultures of *L. lactis* subsp. *lactis* 4 isolates (IMAU11823, IMAU11919, IMAU10987, and IMAU10407) by SPME-GC-MS.

Number	Compound	Chemical formula	RT (min)	RI	RI reference	Abundance (μg/L)			
						
						IMAU10987	IMAU10407	IMAU11823	IMAU11919
**Acid**									
1	Acetic acid	C2H4O2	2.77	635.75	638.00	0.00	0.00	0.00	0.49
2	Pentanoic acid, 3-methyl-	C6H12O2	6.65	803.63	NS	0.00	0.00	0.86	0.00
3	Butanoic acid	C4H8O2	6.97	811.97	820.00	0.52	0.00	0.10	0.00
4	Propanedioic acid, propyl-	C6H10O4	7.18	817.39	NS	0.00	0.00	0.09	0.00
5	Butanoic acid, 3-methyl-	C5H10O2	9.22	871.03	878.00	1.52	0.21	2.23	0.00
6	4-Methyl-2-oxovaleric acid	C6H10O3	12.16	949.58	NS	0.67	13.33	8.73	10.38
7	Hexanoic acid	C6H12O2	14.25	1006.86	999.00	6.22	2.16	0.00	4.39
8	Hexanoic acid, 2-methyl-	C7H14O2	14.25	1006.86	NS	0.00	0.00	2.88	0.00
10	gamma.-Guanidinobutyric acid	C5H11N3O2	20.03	1187.70	NS	0.00	0.00	0.08	0.00
11	Octanoic acid	C8H16O2	20.06	1188.91	1189.00	4.50	0.81	3.82	2.52
**Aldehyde**									
12	Butanal, 3-methyl-	C5H10O	3.47	689.23	689.00	0.00	0.00	37.86	46.87
13	Heptanal	C7H14O	10.59	907.36	907.00	0.00	0.00	0.96	0.00
14	Benzeneacetaldehyde	C8H8O	15.64	1048.71	NS	1.97	0.84	1.25	0.30
15	2-Octenal, (E)-	C8H14O	16.61	1077.78	1067.00	0.00	0.00	1.34	0.00
16	Benzaldehyde, 3,4-dimethyl-	C9H10O	21.01	1221.53	NS	0.00	0.00	0.26	0.00
**Ketone**									
17	2,3-Butanedione	C4H6O2	1.85	STD	MS.STD	1.27	1.86	6.80	7.74
18	2-Butanone, 3-methyl-	C5H10O	2.14	STD	MS.STD	0.00	0.00	0.00	2.50
19	2-Pentanone	C5H10O	3.30	675.78	NS	0.77	0.00	1.32	1.11
20	Acetoin	C4H8O2	4.42	727.83	714.00	1.57	1.14	18.85	16.23
21	2-Heptanone	C7H14O	10.19	896.55	898.00	11.72	6.76	15.84	16.58
22	2-Nonanone	C9H18O	17.28	1098.04	1104.00	5.53	2.86	6.44	6.58
23	2-Undecanone	C11H22O	23.26	1300.83	1305.00	1.01	0.52	1.02	1.28
24	6-Pentadecanone	C15H30O	28.92	1504.60	NS	0.00	0.00	0.30	0.00
**Alcohol**									
25	Cyclohexanol, 4- methyl-, *trans-*	C7H14O	3.51	691.83	NS	0.58	0.00	0.00	0.00
26	1-Butanol, 3-methyl-	C5H12O	4.43	728.21	734.00	52.74	37.02	72.64	67.65
27	1-Hexanol	C6H14O	9.41	876.09	880.00	1.03	0.23	1.67	0.29
28	1-Butanol, 3- methyl-, acetate	C7H14O2	9.73	884.58	NS	0.00	0.00	0.51	0.00
29	1-Heptanol	C7H16O	13.18	977.10	974.00	2.04	0.71	1.94	1.70
30	1-Deoxy-d-mannitol	C6H14O5	13.90	996.57	NS	0.00	0.20	0.00	0.00
31	3-Nonen-1-ol, (E)-	C9H18O	16.60	1077.60	NS	0.00	0.12	1.67	0.88
32	3-Decyn-2-ol	C10H18O	16.61	1077.78	NS	0.00	0.00	0.00	0.68
33	2-Nonen-1-ol	C9H18O	16.61	1077.97	NS	0.00	0.00	0.19	0.00
34	Phenylethyl Alcohol	C8H10O	17.92	1118.78	NS	2.19	1.61	1.26	1.76
35	1-Nonanol	C9H20O	19.75	1178.60	1171.00	0.00	0.00	0.06	0.00
**Ester**									
36	Carbonic acid, methyl pentyl ester	C7H14O3	3.89	709.61	NS	0.00	0.00	0.00	1.78
37	Acetic acid, pentyl ester	C7H14O2	9.74	884.82	NS	0.00	0.00	3.80	0.00
**Hydrocarbon**									
38	n-Hexane	C6H14	2.18	STD	MS.STD	2.84	228.66	1.23	1.29
39	Pentane	C5H12	2.42	609.09	NS	0.00	0.00	0.14	6.84
40	Hexane, 1-chloro-	C6H13Cl	5.48	764.24	NS	0.00	0.30	0.00	0.00
41	Butanoyl chloride, 3-methyl-	C5H9ClO	12.67	963.53	NS	0.00	0.00	0.97	0.00
42	Benzene, 1,2-dichloro-	C6H4Cl2	15.25	1036.88	NS	2.20	0.00	0.00	1.38
43	Benzene, 1,3-dichloro-	C6H4Cl2	15.26	1037.06	NS	0.00	2.39	0.00	0.00
44	Benzene, 1,4-dichloro-	C6H4Cl2	15.48	1043.74	NS	0.00	0.00	98.94	0.00
45	1-Nonyne	C9H16	16.60	1077.42	NS	1.00	0.00	0.00	0.00
46	Benzaldehyde, 3,4-dimethyl-	C9H10O	21.01	1221.53	NS	0.00	0.00	0.26	0.00
**Nitrogen-containing**									
47	Alanine	C3H7NO2	1.42	STD	MS.STD	6.45	1.23	0.53	0.00
48	Dimethylamine	C2H7N	1.52	STD	MS.STD	0.00	0.00	0.32	0.47
49	2-Formylhistamine	C6H9N3O	1.62	STD	MS.STD	0.41	0.00	0.00	0.57
50	D-Alanine	C3H7NO2	1.62	STD	MS.STD	0.00	0.96	1.74	1.03
51	Acetamide, 2-fluoro-	C2H4FNO	2.00	STD	MS.STD	1.82	0.00	0.00	0.00
52	2-Pentanamine, 4-methyl-	C6H15N	2.16	STD	MS.STD	0.00	0.00	3.45	0.00
53	Oxime-, methoxy-phenyl-_	C8H9NO2	11.16	922.82	NS	1.53	0.65	2.25	2.01
54	Benzothiazole	C7H5NS	21.24	1229.54	NS	0.96	0.14	0.90	0.19

*The retention indices (RI) of unknown compounds in the HP-5MS column (Agilent Technologies Inc., Palo Alto, CA, United States) calculated against the GC-MS retention time of n-alkanes (C3–C25). RI from the NIST Chemistry WebBook database (http://webbook.nist.gov/chemistry). The capillary column is shown in parentheses. RI, agrees with retention index literature; MS, compared with the NIST 11 Mass Spectral Database; STD, agrees with the mass spectrum of standard chemicals; –, not detected; NS, not found in any database.*

## Discussion

*Lactococcus lactis* is the most extensively studied LAB and the second most studied gram-positive bacterium with respect to its genetics, physiology and molecular biology (Savijoki et al., 2006; Cretenet et al., 2011). *L. lactis* subsp. *lactis* plays an important role in the food industry, particularly production of dairy products, and in the health sector due to its special fermentation process (Kelleher et al., 2017). It is well known as an industrial starter culture, and is generally recognized as safe (GRAS) by the Food and Drug Administration in United States (Adelene et al., 2017; Kelleher et al., 2017). Some industrial starter culture composed of *L. lactis* subsp. *lactis* have good fermentation capability and flavor, which means they have great economic value. However, there is a need to identify new starter isolates that meet the growing consumer demand for products with a wider variety of flavors and mouthfeel. As one of the main sources of potential starter isolates, traditionally fermented dairy products are abundant in LAB. Therefore, evaluating the fermentation characteristics of *L. lactis* subsp. *lactis* isolates from natural-fermented dairy products helps us understand their metabolic characteristics and identify new applications.

In this paper, we describe the fermentation characteristics of 227 *L. lactis* subsp. *lactis* isolates from traditionally fermented dairy products and identified isolates with potential as starter cultures that had good fermentation capabilities and produced a malt odor. We showed that all 227 *L. lactis* subsp. *lactis* isolates were able to ferment milk within 40 h and that they had a wide diversity of fermentation characteristics. We found that 55 isolates fermented within 12 h, which was very fast, and that a total of 190 isolates had finished fermentation within 32 h. The average speed of acid production in the first 12 h was used to further elucidate acid production capacity and, although this was not linear during fermentation, it was helpful for comparisons amongst isolates. As starter cultures, *L. lactis* subsp. *lactis* are often used in the production of cheese, butter, and various fermented milks; selection has been based on their performance in fermentation and on desired properties of the final product (Wouters et al., 2002). Acidification activity is the most important selection criterion. Isolates that are fast acid producers are most frequently selected as starter cultures, whereas poor or medium acid are only used if they have other desirable attributes (Ehe et al., 2004).

Acid production capacity during storage is also important for the dairy industry. Low post-fermentation acidification is an important selection parameter for commercial starters used in yogurt or fermented milk production as it affects the quality of the final product (Dan et al., 2015). We investigated this in the 190 isolates that had a fermentation time of less than 32 h and measured parameters such as pH, TA, viscosity, WHC and FAN during storage. The isolates exhibited a lot of variation in these attributes during storage which may indicate a high level of genetic diversity, which warrants further study.

The data set on characteristics of *L. lactis* subsp. *lactis* during fermentation and storage that we have compiled will contribute to the further characterization of *L. lactis* subsp. *lactis*. Notably, we found correlations between fermentation characteristics and storage characteristics. The positive correlation between TA during fermentation, and pH and FAN during storage, could indicate that isolates with a higher acid-producing capability during fermentation may result in products with a lower pH during storage, which could cause post-fermentation acidification. This could explain why there were only a few isolates with good fermentation capacities and low post-fermentation acidification, and why the fermentation capacity of most isolates was positively correlated with post-fermentation acidification capacity. At the same time, a rapid decrease in pH may also support protein hydrolysis, which produces more FAN. In addition, proteolytic activity of a isolate affects growth and, ultimately, the sensory quality of the product (Flambard et al., 1998; Ehe et al., 2004). *L. lactis* subsp. *lactis* has a complex proteolytic system that, together with other proteolytic enzymes, converts casein into peptides and amino acids (Law and Haandrikman, 1997). Amino acids are key precursors of volatile flavor compounds once they are metabolized into aldehydes, ketones, amides, alcohols and sulfur compounds (Yvon and Rijnen, 2001; Smit et al., 2005).

It is worth mentioning that, due to the unique metabolic characteristics of *L. lactis* subsp. *lactis*, they produce lactic acid and other products during the fermentation process. This influences microbial safety because it results in a high level of lactic acid and anti-microbial agents such as bacteriocins (Guinane et al., 2005; Bali et al., 2016). Amongst these metabolites, diacetyl is of particular interest. *L. lactis* subsp. *lactis* biovar *diacetylactis* was classified according to its phenotype of diacetyl production. However, the increasing demand for products with a wide range of new organoleptic properties has encouraged collection of big data sets on phenotypic characteristics for this species. Researchers are increasingly screening isolates with the potential to produce different flavors.

Fermentation is a traditional approach to food preservation that improves food safety and also confers enhanced organoleptic, nutritional and health-promoting attributes (Macori and Cotter, 2018). Fermentation also influences flavors, which are chemical sensations produced by particular molecules released from food during consumption (Voilley and Etiévant, 2006). To identify isolates with potential as starter cultures, that will result in fermented milk with a good flavor following industrial production, we focused on the 16 isolates that completed fermentation within 12 h. These 16 isolates underwent sensory evaluation and their fermentation time was re-evaluated. Isolates IMAU11823 and IMAU11919 had the highest sensory scores and faster fermentation speed.

SPME-GC-MS was then used to evaluate the volatile compounds in fermented milk with and without the special flavors; this showed that isolates IMAU11823 and IMAU11919 produced various desirable volatile flavors that were probably the main source of the malt odor (Dan et al., 2017), and included diacetyl, acetoin, 3-methyl butanal and 3-methyl-2-butanone. The compounds 3-methyl butanal and 3-methyl-2-butanone are known to endow fermented dairy products with fresh malt, nutty, floral and fruit flavors (Jensen et al., 1994; Adhikari et al., 2010; Galvão et al., 2011). Because the content of 3-methyl-butyraldehyde was relatively high and its threshold value is lower than 3-methyl butanal and 3-methyl-2-butanone (0.06 μg/mL in aqueous solution), it makes a large contribution to the flavor of nuts (Bertuzzi et al., 2018). Isolates IMAU11823 and IMAU11919 have the greatest potential for use in the industrial production of fermented dairy products, although this needs to be tested for stability and the presence of bacteriophages.

Fermented foods are known to have higher nutritional and functional values compared with their unfermented counterparts (Hasan et al., 2014). As a result, fermentation processes are amongst the most popular food processing techniques to increase nutritional value (Panda et al., 2014). Start cultures, as the soul of fermented food of starter cultures food plays a vital role. Natural dairy production contains the rich resources of LAB and it can continuously provide a wide variety of start cultures isolates for fermentation industry, so from the natural fermented dairy products development of LAB resource is of great significance. Our study found that *L. lactis* subsp. *lactis* in naturally fermented dairy products has extremely rich phenotypic and technological diversity, which deserves further development and utilization.

## Conclusion

In summary the findings from this study contribute to the existing phenotypic data on *L. lactis* subsp. *lactis* fermentation characterization. In addition, we identified two isolates with potential as starter cultures (IMAU11823 and IMAU11919); these isolates had a good fermentation capacity and yielded good sensory profiles. Both isolates produced fermented milk with a good malt and nut flavor; this was as a result of the production of 3-methyl butanal and 3-methyl-2-butanone, as identified by SPME-GC-MS. These two isolates can also be used to study the metabolic mechanism for production of branched aldehydes by *L. lactis* subsp. *lactis*.

## Data Availability Statement

All datasets presented in this study are included in the article/Supplementary Material.

## Ethics Statement

The studies involving human participants were reviewed and approved by the Ethical Committee of the Inner Mongolia Agricultural University. The patients/participants provided their written informed consent to participate in this study.

## Author Contributions

TS and ZS designed the experiments. WL, MR, LD, JL, SW, YS, ML, and WR performed the experiments. WL and QH analyzed the data. WL and JY drafted the manuscript. All authors read and approved the final manuscript.

## Conflict of Interest

The authors declare that the research was conducted in the absence of any commercial or financial relationships that could be construed as a potential conflict of interest.

## Supplementary Material

FIGURE S1 The sample map of all 227 isolates.

TABLE S1 The information table of 227 isolates.

TABLE S2 The criteria of sensory evaluation.
