# Supplementary figures and images for: Fermentation Characteristics of Lactococcus lactis subsp. lactis Isolated From Naturally Fermented Dairy Products and Screening of Potential Starter Isolates
Source: Front Microbiol. 2020 Aug 4;11:1794. doi: 10.3389/fmicb.2020.01794 (PMC7438938; doi:10.3389/fmicb.2020.01794)

1000 km

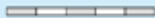

600 mi

⑤

②

⑤

⑨

③

⑥

⑤

⑪

⑫

⑬

③

①

⑫

②

2000 km (equat.)

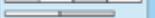

1000 mi (equat.)

Supplement: FIGURE S1 — The sample map of all 227 isolates. [file Data_Sheet_1.PDF]
