# Supplementary material for: Fermentation Characteristics of Lactococcus lactis subsp. lactis Isolated From Naturally Fermented Dairy Products and Screening of Potential Starter Isolates
Source: Front Microbiol. 2020 Aug 4;11:1794. doi: 10.3389/fmicb.2020.01794 (PMC7438938; doi:10.3389/fmicb.2020.01794)
Supplement: TABLE S2 — The criteria of sensory evaluation. [file Table_2.DOCX]

TABLE S2 Sensory evaluation standard

| Index | standard | score |
| --- | --- | --- |
| Flavor（30） | It has a special aroma of fermented milk and a strong aroma | 25-30 |
|  | It has a special aroma of fermented milk and aroma | 20-24 |
|  | No fermented milk with aroma or slightly odor | <20 |
| Taste（30） | Moderate sweet and sour, coordinated | 25-30 |
|  | Sour or sweet, no odor and noisy | 20-24 |
|  | Sour and sweet are not coordinated, there is a small amount of odor | <20 |
| Appearance（20） | No air bubbles, uniform curd, smooth appearance, no whey precipitation / uniform color, milky white or slightly creamy yellow | 15-20 |
|  | No air bubbles, uniform curd, slightly rough appearance, a small amount of whey precipitation / uniform color, yellowish or light yellow | 10-14 |
|  | Bubbles, rough curd, whey precipitation or delamination/color uniformity, slightly grayish yellowish | <10 |
| Feel（20） | Even and delicate taste | 15-20 |
|  | The taste is not delicate enough | 10-14 |
|  | Rough taste | <10 |
